# Complex coevolutionary history of symbiotic Bacteroidales bacteria of various protists in the gut of termites

**DOI:** 10.1186/1471-2148-9-158

**Published:** 2009-07-09

**Authors:** Satoko Noda, Yuichi Hongoh, Tomoyuki Sato, Moriya Ohkuma

**Affiliations:** 1 Ecomolecular Biorecycling Science Research Team, RIKEN Advanced Science Institute, Wako, Saitama 351-0198, Japan; 2 Microbe Division/Japan Collection of Microorganisms, RIKEN Bioresource Center, Wako, Saitama 351-0198, Japan; 3 Current address: Interdisciplinary Graduate School of Medicine and Engineering, University of Yamanashi, Yamanashi 400-8510, Japan; 4 Current address: Graduate School of Bioscience and Biotechnology, Tokyo Institute of Technology, Tokyo 152-8550, Japan; 5 Current address: Research Institute of Genome-based Biofactory, Advanced Industrial Science and Technology (AIST), Hokkaido 062-8517, Japan

## Abstract

**Background:**

The microbial community in the gut of termites is responsible for the efficient decomposition of recalcitrant lignocellulose. Prominent features of this community are its complexity and the associations of prokaryotes with the cells of cellulolytic flagellated protists. Bacteria in the order Bacteroidales are involved in associations with a wide variety of gut protist species as either intracellular endosymbionts or surface-attached ectosymbionts. In particular, ectosymbionts exhibit distinct morphological patterns of the associations. Therefore, these Bacteroidales symbionts provide an opportunity to investigate not only the coevolutionary relationships with the host protists and their morphological evolution but also how symbiotic associations between prokaryotes and eukaryotes occur and evolve within a complex symbiotic community.

**Results:**

Molecular phylogeny of 31 taxa of Bacteroidales symbionts from 17 protist genera in 10 families was examined based on 16S rRNA gene sequences. Their localization, morphology, and specificity were also examined by fluorescent in situ hybridizations. Although a monophyletic grouping of the ectosymbionts occurred in three related protist families, the symbionts of different protist genera were usually dispersed among several phylogenetic clusters unique to termite-gut bacteria. Similar morphologies of the associations occurred in multiple lineages of the symbionts. Nevertheless, the symbionts of congeneric protist species were closely related to one another, and in most cases, each host species harbored a unique Bacteroidales species. The endosymbionts were distantly related to the ectosymbionts examined so far.

**Conclusion:**

The coevolutionary history of gut protists and their associated Bacteroidales symbionts is complex. We suggest multiple independent acquisitions of the Bacteroidales symbionts by different protist genera from a pool of diverse bacteria in the gut community. In this sense, the gut could serve as a reservoir of diverse bacteria for associations with the protist cells. The similar morphologies are considered a result of evolutionary convergence. Despite the complicated evolutionary history, the host-symbiont relationships are mutually specific, suggesting their cospeciations at the protist genus level with only occasional replacements.

## Background

In phylogenetically basal termite species, the so-called lower termites, the gut community comprises several species of flagellated protists (single-cell eukaryotes) as well as a diverse array of prokaryotes [1]. The relationship between termites and cellulolytic protists in their gut is a well-known example of symbiosis; gut protists are essential for the survival of termites that thrive on cellulosic matter [2]. Gut protists of termites belong to either the phylum Parabasalia or the order Oxymonadida (phylum Preaxostyla) and most of them are unique to termites and related wood-feeding cockroaches of the genus *Cryptocercus *[3,4]. They are very difficult to cultivate, and their molecular phylogeny has been studied without cultivation [5-15]. They are likely to have been inherited from a common ancestor of termites and *Cryptocercus *and diversified within the gut [16].

Gut bacteria of termites aid in efficient digestion and a large majority of them comprise yet-uncultivated species [17]. Culture-independent studies indicate that the gut community harbors hundreds of bacterial species most of which are novel [18-23]. Most of the gut bacteria are unique and indigenous to termites, and they phylogenetically form many lineages that comprise bacteria species from diverse termites [24-26]. Methanogenic archaea also inhabit termite guts, but they represent only a small population in the community [27-29]. Albeit this complexity, the composition of the gut microbial community is significantly conserved within a termite genus. The direct transfer of gut fluids (nutrients) from anus to mouth between nest mates (proctodeal trophallaxis), allows the stable vertical transmission of gut symbionts from generation to generation [30].

Most gut protists, if not all, harbor prokaryotes as either endosymbionts that exclusively live within the host protist cells or ectosymbionts that firmly attach onto the cell surface of the host protists. These are characteristic features in the termite-gut community [1,31,32]. Endosymbiotic methanogens [33-35], ectosymbiotic spirochetes [35-38], endo- and ectosymbiotic Bacteroidales members [37,39-45], and endosymbiotic bacteria in the candidate phylum Termite Group 1 (TG1) [46-48] have been reported in several gut protist species. It has been noted that protist-associated bacterial species are one of the predominant populations in termite guts. In one marked example, a single endosymbiont species was shown to account for 70% of the bacterial cells in a termite gut [40]. Therefore, they should considerably contribute to gut metabolism and play important roles [1].

Among these protist-associated bacteria, members in the order Bacteroidales are widely spread in a variety of protist species. At least three distinct lineages in Bacteroidales have been reported as ectosymbionts of seven genera of gut protists [41]. Since then, there have been a growing number of identifications of Bacteroidales members as either ectosymbionts or endosymbionts of termite-gut protists [40,42-45]. Nevertheless, the overall phylogenetic relationships have not yet been fully examined. These studies are also limited to a narrow range of taxonomic sampling in a broad diversity of gut protists and some important protist taxa still lack the identification of associated prokaryotes. Furthermore, morphological appearances of the Bacteroidales associations vary due to differences in cell shape, attachment mode, and the effect on the host protist cell, providing an attractive model to investigate morphological evolution of protist-bacteria associations. Recently, cospeciations of the tripartite symbiotic partners have been described in gut protists of the genus *Pseudotrichonympha*, their Bacteroidales endosymbionts, and the host termites [43]. In general, such coevolutionary studies have usually been conducted with relationships involving endosymbionts [49-54], and few have investigated coevolution with ectosymbionts.

In this study, we investigated Bacteroidales symbionts in a variety of gut protist species, particularly in the protist groups that have been yet-untouched in this regard. Even in the previously examined protist genera, we analyzed multiple species to evaluate the specificity and stability of the symbionts. The phylogeny of the Bacteroidales symbionts were inferred to understand the relationships to the host protist phylogeny, the connection with their morphology, the extent of their specificity, and the evolutionary process of these impressive symbiotic associations.

## Results and discussion

### 16S rRNA gene sequences of symbionts

**Figure 1:**
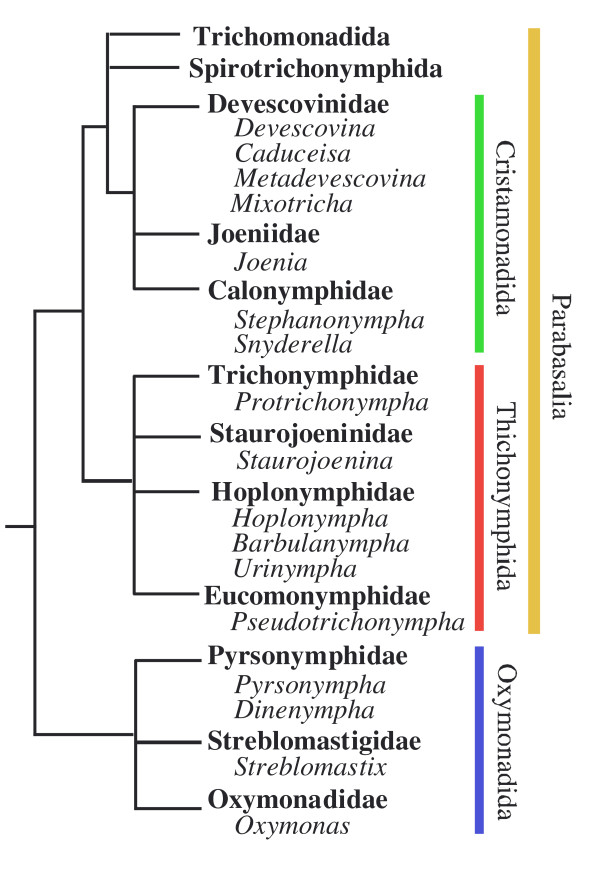
**A schematic tree showing phylogenetic relationships of gut protist families**. The simplified tree was drawn based on their current molecular phylogeny [10-15]. The names of protist families are shown in bold and the genera examined for their Bacteroidales symbionts in this study and previously are listed in each family. The orders Cristamonadida, Trichonymphida, and Oxymonadida are indicated by vertical bars in green, red, and blue, respectively. The range of the phylum Parabasalia is also shown by a vertical bar. No stable association of Bacteroidales members was observed for protist genera in the orders Trichomonadida and Spirotrichonymphida.

Figure 1 outlines the protist genera investigated in this study and their taxonomic assignments. First, the associations of Bacteroidales bacteria with gut protists were surveyed in various species of termites by fluorescent in situ hybridization (FISH) using a group-specific probe for this order. Among the four orders of Parabasalia, the association of Bacteroidales symbionts was rarely observed in members of Trichomonadida and Spirotrichonymphida. Therefore, we focused on the symbionts of three protist orders: Cristamonadida and Trichonymphida (in Parabasalia), and Oxymonadida.

**Figure 2:**
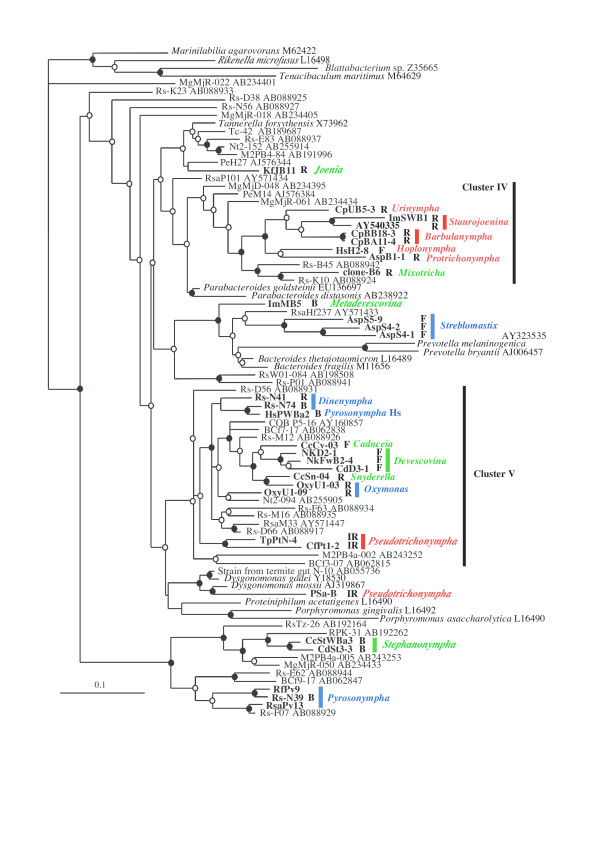
**Phylogenetic relationships of Bacteroidales symbionts of gut protests**. The sequences identified from the symbionts of gut protists are shown in bold letters. Their morphological types and names of their host protist genera are shown after the names of the sequences. Abbreviations of the morphological types examined by FISH or predicted by previous electron microscopy are R, rod-shaped ectosymbionts; F, filamentous ectosymbionts; B, bristle-like ectosymbionts; and IR, intracellular rods (endosymbiotic). The host genera belonging to Cristamonadida, Trichonymphida, and Oxymonadida are shown in green, red, and blue, respectively. Vertical black bars indicate previously described clusters of termite-gut Bacteroidales members (clusters IV and V) [19]. Nodes supported by both an ML-bootstrap value of > 70% and a Bayesian posterior probability of > 95% are indicated by filled circles. Those of > 50% supported by either ML-bootstrap or Bayesian analysis are represented by open circles. Reference sequences tagged Rs, Rsa, and RPK were identified from whole gut communities of *Reticulitermes *termites, whereas those tagged Nt, M2, Mg, Tc, COB, and BCf were identified from the termite genera *Nasutitermes*, *Microcerotermes*, *Macrotermes*, *Termes*, *Cubitermes*, and *Coptotermes*, respectively. Reference sequences tagged Pe were identified from the gut community of a scarab beetle of the genus *Pachnoda*. Scale bar represents 0.1 nucleotide substitutions per position.

The cells of the protist species that stably harbored Bacteroidales symbionts were physically isolated and used for PCR amplification of bacterial 16S rRNA gene. Multiple clones in each protist species were sorted into phylotypes with a criterion of > 98% sequence identity. In most cases, a single phylotype belonging to the order Bacteroidales was the most abundant in clone numbers. Although the FISH detected stable associations of Bacteroidales symbionts with the protist species in the genera *Stephanonympha *and *Pyrsonympha*, [36,38], we failed to identify any Bacteroidales-like sequence from these protist species with bacterial universal PCR primers because numerous spirochetes attached onto their cell surface; however, we successfully used primers that could not amplify most spirochete sequences to identify their Bacteroidales symbiont sequences. As a whole, we identified the phylotype sequences of the Bacteroidales 16S rRNA gene from 11 protist species of 9 genera, and together with previously reported sequences, 31 sequences were phylogenetically examined (Table 1 and Figure 2). This range of taxon sampling covered important lineages of host protist species in the gut of termites and *Cryptocercus *cockroaches.

**Table 1:** The sequences of Bacteroidales symbionts identified from gut protist species.

Clone name	Protist species	Host insect	Accession number	References
NkFWB2-4	*Devescovina *sp. NkFWS	*Neotermes koshunensis*	AB462742	This study
NkD2-1	*Devescovina *sp. Nk2	*Neotermes koshunensis*	AB194938	[41]
CdD3-1	*Devescovina lemniscata*	*Cryptotermes domesticus*	AB194939	[41]
CcCv-03	*Caduceia versatilis*	*Cryptotermes cavifrons*	AB299517	[45]
ImMB5	*Metadevescovina cuspidata*	*Incisitermes minor*	AB462743	This study
B6	*Mixotricha paradoxa*	*Mastotermes darwiniensis*	AJ488195	[37]
KfJB11	*Joenia annectens*	*Kalotermes flavicollis*	AB462744	This study
CcStWBa3	*Stephanonympha *sp.	*Cryptotermes cavifrons*	AB462745	This study
CdSt3-3	*Stephanonympha *sp.	*Cryptotermes domesticus*	AB462746	This study
CcSn-04	*Snyderella *sp.	*Cryptotermes cavifrons*	AB462747	This study
AspB1-1	*Protrichonympha *sp.	*Archotermopsis *sp.	AB462749	This study
ImSWB1	*Staurojoenina assimilis*	*Incisitermes minor*	AB462748	This study
--^a^	*Staurojoenina *sp.	*Neotermes cubanus*	AY540335	[39]
HsH2-8	*Hoplonympha *sp.	*Hodotermopsis sjoestedti*	AB194940	[41]
CpBB18-3	*Barbulanympha *sp.	*Cryptocercus punctulatus*	AB200973	[41]
CpBA11-4	*Barbulanympha ufalula*	*Cryptocercus punctulatus*	AB200972	[41]
CpUB5-3	*Urinympha talea*	*Cryptocercus punctulatus*	AB200971	[41]
CfPt1-2	*Pseudotrichonympha grassii*	*Coptotermes formosanus*	AB218918	[40]
TpPtN-4	*Pseudotrichonympha *sp.	*Termitogeton planus*	AB218919	[40]
PSa-B	*Pseudotrichonympha *sp.	*Psammotermes allocerus*	AB262561	[43]
HsPWBa2	*Pyrsonympha *sp. 3	*Hodotermopsis sjoestedti*	AB462750	This study
Rs-N39	*Pyrsonympha grandis*	*Reticulitermes speratus*	AB088920	This study
RsaPv13	*Pyrsonympha vertens*	*Reticulitermes santonensis*	AY572027	[46]
RfPv9	*Pyrsonympha vertens*	*Reticulitermes flavipes*	AY572026	[46]
Rs-N41	*Dinenympha *spp.	*Reticulitermes speratus*	AB088947	This study
Rs-N74^a^	*Dinenympha *spp.	*Reticulitermes speratus*	AB088917	[44]
Asp4-1	*Streblomastix *sp.	*Archotermopsis *sp.	AB194942	[41]
Asp4-2	*Streblomastix *sp.	*Archotermopsis *sp.	AB194943	[41]
Asp5-9	*Streblomastix *sp.	*Archotermopsis *sp.	AB194945	[41]
NkOxy1-9	*Oxymonas *sp.	*Neotermes koshunensis*	AB231290	[42]
NkOxy1-3	*Oxymonas *sp.	*Neotermes koshunensis*	AB231289	[42]

^a ^Candidate epithets, 'Vestibaculum illigatum' and 'Symbiothrix dinenymphae', were given for the symbionts of *Staurojoenina *and *Dinenympha*, respectively.

### FISH identifications

**Figure 3:**
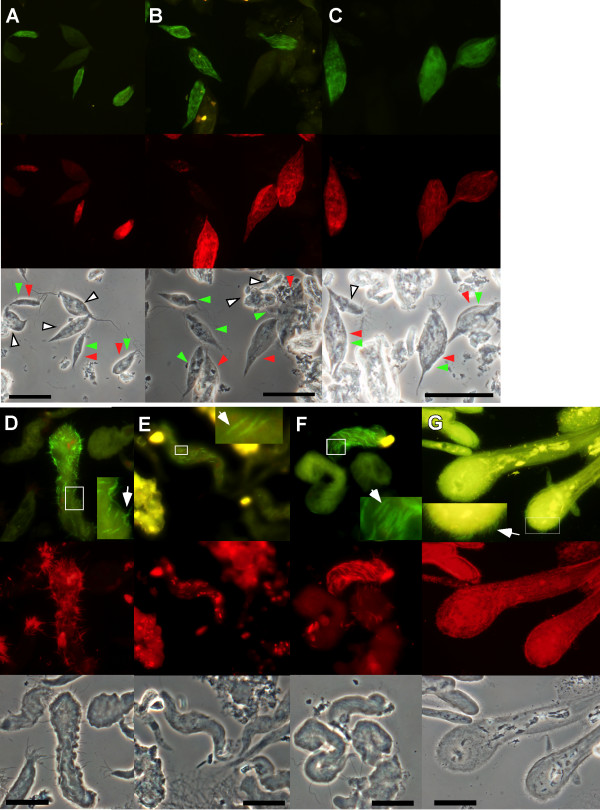
**In situ detection of Bacteroidales symbionts of gut protists of termites**. (A-C) Ectosymbionts of *Devescovina *spp. in the gut of termite *Neotermes koshunensis*. (A) Ectosymbionts corresponding to the sequence NkFWB2-4 (labeled with 6FAM, stained in green) were associated with the host protist species represented by the sequence NkFWS (labeled with Texas Red, stained in red). (B) A mixture of *Devescovina *spp. cells was simultaneously hybridized with the sequence-specific probes for NkFWB2-4 (green) and NkD2-1 (red). (C) Ectosymbionts corresponding to the sequence NkD2-1 (red) were detected in the host protist species represented by the sequence Nk2 (green). Arrowheads in phase-contrast images of A-C indicate the stained *Devescovina *cells in colors corresponding to the probes, and the cells not detected by either probe are indicated by white arrowheads. (D-F) Detection of the ectosymbionts of protists in the genera *Pyrsonympha *and *Dinenympha *in the gut of *R. speratus*. (G) Detection of the ectosymbionts of *Pyrsonympha *sp.3 in the gut of *H. sjoestedti*. The upper panels in D-G show images obtained using sequence-specific probes labeled with 6FAM (green), and the middle panels show images obtained with the general bacterial probe (red). Insets in the upper panels of D-G are magnifications of the images indicated by squares. Arrows in panels D-G indicate typical ectosymbionts. Amorphous yellow signals in the upper panels and the corresponding signals in the middle panels were probably derived from autofluorescence of ingested wood particles. Scale bars: 50 μm (A-C, G) and 20 μm (D-F).

The localization, morphology, and specificity of protist-associated Bacteroidales symbionts were examined by FISH. In a previous report, the ectosymbiont corresponding to the phylotype NkD2-1 was identified from the largest cells of the genus *Devescovina *in the termite *Neotermes koshunensis *[41]. In this termite, however, there are at least three *Devescovina *species represented by distinct sequences of eukaryotic small subunit rRNA gene [5,7]. Another Bacteroidales phylotype, NkFWB2-4, was identified in this study from a smaller *Devescovina *cell represented by the protist sequence NkFWS. FISH using a specific probe for phylotype NkFWB2-4 always gave positive signals in the symbionts of *Devescovina *cells stained with a specific probe for the host sequence NkFWS (Figure 3A). Sequence-specific FISH also showed that the symbiont NkD2-1 exclusively associated with *Devescovina *sp. corresponding to the protist sequence Nk2 (Figure 3C). Simultaneous detections of the symbionts NkFWB2-4 and NkD2-1 gave positive FISH signals from completely separate protist cells (Figure 3B). Each combination of the probes for (1) the host Nk2 and the symbiont NkFWB2-4, (2) the host NkFWS and the symbiont NkD2-1, and (3) the hosts Nk2 and NkFWS did not give FISH signals in the same host protist cells (data not shown). The other *Devescovina *sp. represented by the sequence Nk9 harbored Bacteroidales symbionts detected with a group-specific probe for cluster V of Bacteroidales [41], but the probes for neither NkFWB2-4 nor NkD2-1 detected them (data not shown), indicating the presence of the third yet-unidentified symbiont species specific to *Devescovina *sp. Nk9. The results indicate that each of the three *Devescovina *spp. in *N. koshunensis *harbored the specific Bacteroidales symbionts.

Oxymonad protist species of the genera *Pyrsonympha *and *Dinenympha *(Pyrsonymphidae) exist in a high cell number in the termite *Reticulitermes speratus *[8,55]. In this study, the phylotype of the Bacteroidales symbionts was examined in *Pyrsonympha grandis*, *Dinenympha exilis*, and *Dinenympha porteri*. The identified Bacteroidales sequences were assigned to phylotypes Rs-N39, Rs-N41, and Rs-N74, respectively, which were previously obtained from the whole gut community of this termite [20]. The Rs-N74 phylotype has recently been described as an ectosymbiont of *Dinenympha *spp. [44]. Sequence-specific probes for Rs-N39 and Rs-N41 were successfully applied in the FISH detections of the corresponding ectosymbionts, and the specific signals were obtained for Rs-N39 in *P. grandis *(Figure 3D) and for Rs-N41 in *Dinenympha *spp. including *D. exilis *(Figure 3E and 3F). FISH using a probe for the sequence identified from the largest *Pyrsonympha *species (designated sp.3 [56]) in the termite *Hodotermopsis sjoestedti *gave signals specifically in its ectosymbionts (Figure 3G).

The Bacteroidales symbionts of *Protrichonympha *sp. were also detected by FISH using a sequence-specific probe (data not shown), while only the group-specific probe for Bacteroidales was applied for the detection of the symbionts of the other protist species.

### Host-symbiont specificity and consistency

Most of the Bacteroidales symbionts were not shared between each protist species even when the congeneric protist species coexisted in the same termite gut as clearly shown by the ectosymbionts of *Devescovina *spp. in the gut of *N. koshunensis*. Each of the specific probes examined so far usually gave FISH signals from symbionts of only a single protist species, indicating a high specificity of the association. This mutual specificity between Bacteroidales symbionts and their hosts is in clear contrast to the cases of ectosymbiotic spirochetes and endosymbiotic methanogens. A single spirochete species is often shared by the host protists in different genera [36,38] and closely related endosymbiotic methanogens are often shared between distantly related protist species [33,34]. Therefore, the host-symbiont specificity is lower in spirochetes and methanogens than in Bacteroidales bacteria.

A unique phylotype of the Bacteroidales symbionts was usually identified in a protist species, which is also in clear contrast to ectosymbiotic spirochetes; a single protist cell usually harbors multiple spirochete species [36,38]. Although the presence of the second associated Bacteroidales species cannot be denied completely, the FISH using the probe for the Bacteroidales group detected the symbionts of only the similar morphology. Therefore, the identified sequence from each protist species is considered to represent the most abundant symbiont species. Meanwhile, simultaneous associations of bacteria in distinct phyla with a single protist cell are common in termite guts, as typically shown by Bacteroidales and spirochete ectosymbionts on the same cells of diverse protist species [38,41,42] and by other examples [45,57].

The specific associations of Bacteroidales symbionts with protists seem to be stable and are consistently observed. The sequence-specific FISH experiments reported in this study and previously indicated that a particular species of protist even in the different individuals of the host termite species almost always harbored the same Bacteroidales symbionts. The FISH using the group-specific probe also detected the symbionts of the same morphology from almost all the cells of the protist species. As previously mentioned [31], bacterial associations of typical morphologies have been often critical for taxonomic classifications of the host protists. Furthermore, numbers of the cells of the Bacteroidales symbionts per host protist cells are always within similar ranges: for instance, several thousands in *Staurojoenina anectens*, *Protrichonympha *sp., and *Pyrsonympha *sp. 3; several hundreds in *Devescovina *spp.; and several tens in *Stephanonympha *spp., *Snyderella *sp., *Metadevescovina *sp., *Dinenympha *spp., and *P. grandis*. These observations indicate that the Bacteroidales associations are not occasional. The specificity and consistency of the Bacteroidales associations strongly suggest the established, stable symbiotic relationship between Bacteroidales bacteria and their host protists.

The exceptions of mutual specificity and consistency of the Bacteroidales associations appeared in the case of the protist order Oxymonadida. FISH using sequence-specific probes for Rs-N74 and Rs-N41 demonstrated that each of the corresponding ectosymbionts was associated with, and was thus shared among, multiple species of *Dinenympha*, although species in this genus are extremely closely related to one another [8,9]. In addition, two morphologically distinct Bacteroidales ectosymbionts were occasionally detected in single cells of *D. porteri*: one was bristle-like and the other was rod-shaped, probably corresponding to the phylotypes Rs-N74 and Rs-N41, respectively (see below). Two distinct Bacteroidales phylotypes have also been identified in a single species of the genus *Oxymonas*, but these two ectosymbiont phylotypes rarely occur simultaneously in individual cells of *Oxymonas *sp. [42]. It is noted that considerable portions of *Oxymonas *sp. and *Dinenympha *spp., and a small portion of *P. grandis *did not harbor any detectable Bacteroidales symbionts, suggesting that the association of Bacteroidales members is not always obligate in these protist species.

### Relationship between host and symbiont

The phylogenetic identifications of Bacteroidales symbionts in a variety of gut protist species revealed that the symbionts were distributed widely in this order of bacteria (see Figure 2). The symbionts did not form a single monophyletic lineage but occurred as multiple lineages. Even within a protist order, the symbionts of different protist genera were phylogenetically diverse. Indeed, approximately unbiased (AU) tests [58] revealed that monophyly of the symbionts of each of the three protist orders was completely rejected (*P *< 0.003). The results suggest that various Bacteroidales species have been independently acquired by gut protists during their evolution.

Analysis of molecular variance (AMOVA) [59] of the symbiont sequence at three hierarchical levels of the host protist taxonomy indicated that on average 70% of the symbiont sequence variation occurred within protist families (namely, among genera), and 28% between families within protist orders (Table 2), which was reflected by the distinct lineages of the symbionts among protist genera. The differences of the symbiont sequence variations were statistically significant within families and among families within the orders (Table 2).

**Table 2:** AMOVA of the Bacteroidales symbiont sequences at three hierarchical levels of their host protist taxonomy.

Source of variation	d.f.	Sum of Squares	Variance components	Percentage of variation	Fixation indices	*P*-value
Among orders	2	0.374	0.00153	1.65	**F_CT_**: 0.01647	0.25709
Among families within orders	7	0.956	0.02635	28.28	**F_SC_**: 0.28757	< 0.0001
Within families	21	1.371	0.06528	70.07	**F_ST_**: 0.29930	< 0.0001
Total	30	2.701	0.09316	100		

In contrast to the distinct symbiont lineages among the host protist genera, the symbionts of many of the congeneric protist species were closely related and formed monophyletic lineages. These protist genera were *Devescovina*, *Stephanonympha*, *Staurojoenina*, and *Barbulanympha*. The monophyletic grouping also occurred in the symbionts of *Devescovina *and *Caduceia *in cluster V of Bacteroidales and these two protist genera are phylogenetically closely related [12]. These close relationships of the ectosymbionts between the related protist species suggest that the symbionts were acquired before diversification of these protist species. A close monophyletic relationship of the Bacteroidales endosymbionts and the cospeciation with their host protists have been demonstrated in 13 of the 14 taxa of the protist genus *Pseudotrichonympha *(represented by the sequences CfPt1-2 and TpPtN-4) with only one obvious exception (sequence PSa-B) [43]. Another exception was the distant phylogenetic relationship between the ectosymbionts HsPWBa2 and Rs-N39 of the protist genus *Pyrsonympha*. The symbiont HsPWBa2 of *Pyrsonympha *sp.3 was rather closely related to the symbiont Rs-N74 of *Dinenympha *spp. The gut protist compositions in the host termites *H. sjoestedti *and *R. speratus *are very similar and the horizontal transfer of gut microbita has been hypothesized [60]. The symbiont Rs-N39 of *P. grandis *was related to the sequences obtained from *Pyrsonympha vertens *(sequences RfPv9 and RsaPv13 [22,46]).

A large monophyletic group occurred in cluster IV of Bacteroidales, which comprised symbionts of related protist species in the three families Hoplonymphidae, Staurojoeninidae, and Trichonymphidae of the order Trichonymphida. One explanation for this monophyletic group is that the symbiont was once acquired by a common ancestor of these three protist families and stably transmitted to their offspring; however, the branching orders of the symbionts were not congruent with their host protist phylogeny (see Figure 2). Also, this monophyletic lineage was distantly related to the symbionts (endosymbionts) of *Pseudotrichonympha*, the other protist genus in Trichonymphida examined so far.

The symbionts of *Devescovina *and *Caduceia *protists formed a monophyletic group together with the symbionts of the distantly related protists genera *Snyderella *and *Oxymonas*, and interestingly, these protist genera exclusively occurred in the termite family Kalotermitidae. For these symbionts, host-switches or replacements might have occurred within the gut microbial community. The taxonomy of the host termites might have affected the protist-symbiont relationships, although AU tests indicated that monophyly of the symbionts in each host termite family (Kalotermitidae, Termopsidae, or Rhinotermitidae) was completely rejected (*P *< 0.001).

### Morphology of the associations

The Bacteroidales symbionts associated with the gut protists show various morphological appearances. Among the ectosymbionts of the protists in Hoplonymphidae, Staurojoeninidae, and Trichonymphidae, an elongated filamentous form aligning in rows along the longitudinal direction of the host protist cell occurred only in *Hoplonympha *sp. [41,61]; the other symbionts in these three protist families were short rods [39,41,62-64]. Filamentous ectosymbionts with similar association occur in *Devescovina *[41,65], *Caduceia *[45,66], and *Streblomastix *[41,67]. Therefore, these filamentous forms have arisen independently. The cells of *Hoplonympha *and *Streblomastix *share similar morphological features displaying several longitudinal deep furrows in their cells and thin, outwardly extending vane-like structures in the transverse sections [41,61,67], allowing the attachment of numerous ectosymbionts. As discussed previously [41], these impressive appearances are likely evolutionary convergent because both the host protists and the symbionts are distantly related to each other.

Ectosymbionts with a bristle-like appearance have recently been described [44], in which the elongated symbiont cells are associated with the host protist at their tip, similar to ectosymbiotic spirochetes. This form of ectosymbionts occurred in the protist genera *Dinenympha *[44], *Metadevescovina *[44], *Pyrsonympha *(see Figure 3G), and *Stephanonympha *(data not shown). Their phylogenetic identifications in distinct lineages suggest that they have arisen by evolutionary convergence with multiple origins. These bristle-like ectosymbionts are usually scattered among numerous ectosymbiotic spirochetes in the same protist cells, suggesting that this form is important for sharing spatial niches with spirochetes.

Phagocytosis of the ectosymbionts by the host protist cells is observed in large numbers in *Barbulanympha *[62] and *Protrichonympha *(data not shown), and to a lesser extent in many other protist species. The phagocytosed bacteria appear to be surrounded by a membrane of the host protists and they maintain their attachment junction with the surrounding membrane. These observations imply that the phagocytosis of ectosymbionts within the protist cell has led to stable intracellular endosymbiosis as discussed previously [62]; however, the lineages of endosymbionts of *Pseudotrichonympha *were completely distinct from the other ectosymbiont lineages examined so far (Figure 2).

AU tests completely rejected monophyly of each of the rod-shaped, bristle-like, and filamentous forms (*P *< 0.001). Monophyly of the endosymbionts was also rejected (*P *= 0.024). AMOVA revealed that genetic variation was more pronounced within similar types of morphology (87%) than among different morphologies (13%).

### Implication of the origin and functional interaction

Gut bacteria in general form several phylogenetic clusters unique to termites, and these clusters usually contain taxa from multiple termite species [19,24-26]. The lineages of the Bacteroidales symbionts of the gut protists are frequently located in these clusters. Many members of these clusters are considered free-swimming in the gut fluid or associated with the gut wall. The gut wall-associated members are clearly shown to be distributed widely in Bacteroidales including clusters IV and V [68,69]. Interestingly, the ectosymbiont sequence of *Mixotricha *in cluster IV of Bacteroidales was closely related to the sequence Rs-K10 identified as representing a species associated with the gut wall [37,69]. These observations imply that gut protists have acquired their Bacteroidales symbionts from a pool of the gut bacteria, which are largely comprised of an indigenous population to termites but may contain provisionally inhabiting species taken up by termites from the surrounding environment.

The acquisition of multiple lineages of Bacteroidales symbionts suggests their common function required for symbiotic associations with gut protists. Although several functions of the Bacteroidales symbionts have been speculated [32,41], the complete genome sequences of two endosymbiont species of gut protists, one belonging to the candidate phylum TG1 [70] and the other to the Bacteroidales order (represented by the sequence CfPt1-2 in cluster V) [71], have disclosed their definitive roles. Provision of essential nitrogenous nutrients such as amino acids and cofactors to the host protists is a common role inferred for the endosymbionts, and the host protists likely supply sugars produced by cellulose degradation to the endosymbionts. Because wood fed on by termites is poor in nitrogen, this role of the endosymbionts is reasonably crucial for nutrition of host protists as well as host termites. The Bacteroidales ectosymbionts are also considered to play a similar role and many are indeed phagocytosed by the host protists as described above. Although further study is necessary to demonstrate their exact functions, the spectrum of required nutrients is probably different among gut protist species and the protists might have selected their suitable partner from the gut bacterial population that harbors diverse metabolic abilities. In the case of the CfPt1-2 symbiont species, the potential abilities for nitrogen fixation and recycling of the putative nitrogen wastes of the host protists further reinforce the efficient provision [71], and the presence of these abilities should also be addressed in the Bacteroidales ectosymbionts.

## Conclusion

Extensive sampling of the sequences of Bacteroidales symbionts of gut protists revealed a more comprehensive view of the phylogenetic complexity of host-symbiont relationships, caused by multiple independent acquisitions of the symbionts. At least in an evolutionary sense, the presence of diverse gut bacteria that potentially harbor various metabolic abilities is advantageous for the host protists to acquire their suitable partner. The acquisitions might be necessary for their mutual interactions depending on both partners, and their functional interactions should be clarified to understand the complex evolutionary history of the associations. Considering that gut protists and their associated bacteria are major populations in termite guts [1,72], the functional interactions are also important to understand how the gut microbial community works for efficient utilization of recalcitrant lignocellulose. Furthermore, the FISH experiments enabled us to characterize the symbionts morphologically and to evaluate their specificity and distribution. The morphological variations observed for the Bacteroidales associations have likely originated from evolutionary convergence and these variations might be advantageous to establish their spatial niches or habitats on the host protist cells, which are sometimes shared with other groups of bacteria. Temporal associations of some Bacteroidales lineages having similar physiological features with certain protist species and replacements of the symbionts cannot be completely rejected; if these events happen even in present days, the symbiotic associations are still in dynamic processes possibly toward more effective digestion in the gut community. However, despite the complex acquisition of symbionts and their convergent evolution, most of the host-symbiont relationships are apparently specific and consistent, suggesting stable vertical transmissions of the symbionts at every cell division of host protists. The fact that congeneric protist species mostly harbor closely related symbionts also supports cospeciations of the Bacteroidales symbionts with their host protists. Once both partners have met, they might gain advantages through adaptation and specialization to establish stable and efficient relationships.

## Methods

### Data collection

The protist species used in this study and their host termites are listed in Table 1. Living termites or specimens preserved in acetone were used. Each protist species was identified based on the morphological characteristics [55,56,61,64,66,73]. The cells of protist species showing the typical morphology were physically isolated by using a micromanipulator (Eppendorf Tranferman NK2) and were extensively washed as described previously [35]. The isolated protist cells were used for PCR amplification of the 16S rRNA gene of the symbiotic bacteria as described previously [41]. Previously described PCR primers universal for bacteria [41] were usually used, but in the cases of protist species in the genera *Stephanonympha *and *Pyrsonympha*, a newly designed primer Bacte3'R (5'-GGAYRTAAGGGCCGTGCT-3') instead of the universal bacterial primer for the 3'-side were used. Bacte3'R covers most of the Bacteroidales members but has mismatches against the sequences of spirochetes and bacteria of the TG1 phylum. The PCR products were cloned into the pCR2.1-TOPO vector (Invitrogen). Partial DNA sequences of multiple clones (15–24 clones except 11 clones for the case of *Devescovina *sp. NkFWS) were determined and sorted. The entire DNA sequence was determined in each representative clone using sequencing primers described previously [42]. In the case of *Devescovina *sp. NkFWS, a physically isolated single cell was subjected to isothermal whole genome amplification (WGA) as described previously [45], and the amplified DNA was used as a template for PCR. The similar methods for physical separation of protist cells have successfully applied both for the identification of their associated prokaryotes in a variety of taxonomic groups and for the gene analyses of protists themselves [8,9,11-14,16,33,35-48,57,74], and in most cases, the origins of the identified sequences have been confirmed by sequence-specific in situ hybridizations. Indeed, using the same isolation method, we analyzed associated bacteria of the protist species of *Dinenympha rugosa *that could not detect any Bacteroidales association by FISH, and no Bacteroidales-like sequence were identified although sequences belonging to other groups of bacteria were obtained. The sequences reported in this study have been deposited in the database [DDBJ: AB462742–AB462750].

### Fluorescent in situ hybridization (FISH)

FISH experiments for the detection of ectosymbionts were performed as described previously [38]. The probe Bactd-937 for most Bacteroidales members [23] was used for the initial survey of protist-associated symbionts. The previously reported general bacterial probe [36] and the probes that detect most spirochete cells in termite guts [38] were used as controls for cell permeability. Specific probes for the protist-associated symbionts designed in this study were 5'-GGCACCCCTGTTGCATCC-3' for the sequence NkFWB2-4, 5'-CGCATTTTATCCCCCTGCAA-3' for the sequence NkD2-1, 5'-GCGCTATCGGAGTTCTTTATA-3' for the sequence HsPWBa2, 5'-TGACTCCCCTGTGTTATGC-3' for the sequence RsN41, and 5'-CCATAGGACCGTCAATCC-3' for the sequence RsN39. Probes for the detection of protist species were 5'-GGTCCTGCTATCAATAAATAAC-3' for *Devescovina *sp. NkFWS and 5'-GGTCCTGCTATCTTTTTCTTAG-3' for *Devescovina *sp. Nk2. These probes were labeled at the 5'-end with either 6-carboxyfluorescein (6-FAM) or Texas Red.

### Phylogenetic analyses

The 16S rRNA gene sequences identified in this study were aligned using the ARB package [75]. Accession numbers for the reference sequences used in the phylogenetic analysis are indicated in Figure 2. A general time-reversible model with gamma distributed rates and an invariable site-rate category (GTR+I+Γ), selected with Modeltest ver.3.06 [76] as the best-fit model of nucleotide substitution, was used for the phylogenetic inferences by the maximum likelihood (ML) and Bayesian methods. The ML tree was constructed using PHYML v2.4.4 [77]. The robustness of the branching pattern was confirmed by bootstrap analysis of 1000 replicates. Bayesian posterior probabilities were calculated using MrBayes 3.0b4 [78], which was started with a random tree, run for 500,000 generations in four chains and "burn-in" of 100,000 generations to ensure the use of only stable chains.

Alternative tree topologies under monophyletic constraints of the concerned symbiont groups were obtained with Bayesian analyses using only the dataset of the symbionts of gut protists except the sequences RfPv9 and RsaPv13. The Bayesian analyses were performed with 300,000 generations with burn-in at 50,000 generations. Therefore, the resulting constraint trees were merely approximate, but we considered that these approximate estimations were enough to reject some alternative phylogenetic hypotheses. Differences in alternative tree topologies were compared by the AU tests implemented in CONSEL [58] using the site-wise log-likelihood outputs obtained with TREE-PUZZLE 5.2.

### Statistical analysis using AMOVA

AMOVA was conducted with Arlequin ver. 3.1 [59] using uncorrected pair-wise sequence distances as a measure of genetic variances among the symbionts. Significance was assessed through 10,000 random permutation replicates.

## Authors' contributions

SN and MO designed the research and drafted the manuscript. SN, YH and TS performed the experiments and collected data, and SN analyzed the data. All authors have read and approved the final manuscript.
